# Multi-View Metal Parts Pose Estimation Based on a Single Camera

**DOI:** 10.3390/s24113408

**Published:** 2024-05-25

**Authors:** Chen Chen, Xin Jiang

**Affiliations:** Mechanical Engineering and Automation, Harbin Institute of Technology, Shenzhen 518055, China; chenchen92@stu.hit.edu.cn

**Keywords:** RGB perception, deep learning for visual perception, pose estimation

## Abstract

Pose estimation of metal parts plays a vital role in industrial grasping areas. It is challenging to obtain complete point clouds of metal parts because of their reflective properties. This study introduces an approach for recovering the 6D pose of CAD-known metal parts from images captured by a single RGB camera. The proposed strategy only requires RGB images without depth information. The core idea of the proposed method is to use multiple views to estimate the metal parts’ pose. First, the pose of metal parts is estimated in the first view. Second, ray casting is employed to simulate additional views with the corresponding status of the metal parts, enabling the calculation of the camera’s next best viewpoint. The camera, mounted on a robotic arm, is then moved to this calculated position. Third, this study integrates the known camera transformations with the poses estimated from different viewpoints to refine the final scene. The results of this work demonstrate that the proposed method effectively estimates the pose of shiny metal parts.

## 1. Introduction

**Figure 1:**
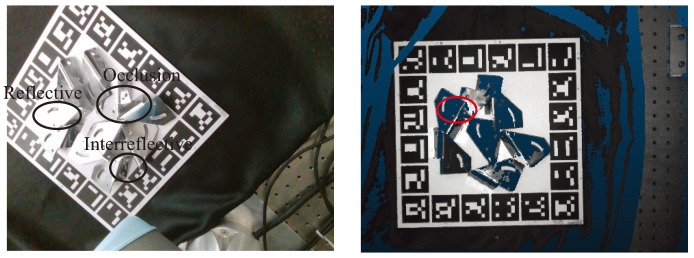
The left figure shows that the metal parts are usually reflective with different appearances. The part marked by red cruve in the right figure shows that the depth information is sometimes missing due to the reflective property.

Pose estimation for metal parts is essential in robot grasping and manipulation areas. The robotic manipulation tasks usually require exact object poses with a global coordinate frame [1,2]. As shown in Figure 1, due to the metal parts’ textureless, reflective, and self-occlusion properties, the current methods can only estimate the poses of some metal parts at first glance. Therefore, this work needs to plan some subsequent viewpoints to estimate the metal parts better. Due to the absence of texture features, the challenge of estimating the 6D pose of textureless objects is often addressed using depth [3,4] or RGB-D information [5,6,7]. These techniques require high-quality depth data.

The RGB-D approaches perform well when the objects are non-reflective [8]. However, pose estimation for shiny and textureless metal parts is challenging because RGB-D cameras usually output fragmented depth images. High-precision point clouds and depth images are typically accessible only through industrial-grade depth cameras or 3D scanners. However, these devices are often relatively expensive. The cheap RGB-D cameras usually cannot get high-precision depth images when sensing shiny metal parts. Therefore, pose estimation of metal parts with RGB images has raised attention [9,10].

The advancement of convolutional neural networks has enhanced pose estimation for textureless metal parts, outperforming traditional methods that rely on local or global features [11]. Nevertheless, RGB-based approaches often exhibit low accuracy in 6D pose estimation due to the inherent difficulty of precisely determining scale and depth from a single viewpoint [10]. Multi-view images containing more information and details yield improved pose estimation results. In light of this, recent studies have utilized multiple RGB images from various viewpoints to enhance the accuracy of object pose estimation [12,13,14]. Although fusing estimated poses from different views can enhance overall performance, this technique still presents challenges in specific scenarios, such as appearance ambiguities and potential occlusions.

This work must address the following issues when jointly estimating the poses of metal parts from multi-views. First, the metal parts’ pose from different views should be expressed in one reference frame. Second, this work must figure out the metal parts’ error of estimation pose in each view. Third, pose estimation in the first view may have some errors, so the proposed method needs a subsequent view for further pose estimation. When transitioning from the current frame to the next frame, this work should choose an appropriate pose to drive the arm for a better additional view of the scene.

## 2. Related Work

This section reviews the techniques employed for 6D pose estimation, mainly focusing on methods using only RGB images.

### 2.1. Single-View 6D Pose Estimation

Many methods have been employed in recent years for textureless object pose estimation with RGB images. Traditional methods usually use template matching methods to estimate textureless object poses [15,16]. However, those methods are generally sensitive to cluttered environments. Furthermore, textureless objects inherently lack surface detail, presenting significant challenges for pose estimation using local features. Moreover, using global templates proves ineffective when these objects are occluded. With the development of convolutional neural networks, some studies employ learned descriptors to estimate pose, like AAE [17]. Some studies employ CNNs to regress predefined 2D keypoints for objects with known models [18,19,20]. These works establish sparse 2D–3D correspondences and subsequently utilize the Perspective-n-Point algorithm to compute the pose [21]. Some work predicts comprehensive 2D–3D correspondences followed by pose computation via 2D–3D correspondences utilizing a PnP method [11,22,23]. Hybridpose [24] extends the PVNet [11], which not only estimates keypoints but also predicts edge vectors and symmetry correspondences. These elements are then collaboratively utilized to estimate the pose.

### 2.2. Multi-View 6D Pose Estimation

Pose estimation employing multi-view methods can enhance the accuracy beyond single-view approaches, especially in occlusions. This improvement is attributable to the capability of these methods to observe objects from multiple perspectives. Traditional methods utilize local features [25], which are ineffective for textureless objects. Recent studies on multi-view pose estimation have seen multiple approaches employing neural networks to tackle this task. MV6D predicts the 6D pose from multiple viewpoints and constitutes an end-to-end approach [26], eliminating the need for multiple prediction stages. Nevertheless, it relies on RGB-D data. Kaskman [27] employs synthetic data to estimate the pose with multiple RGB frames. CosyPose [28] performs effectively by utilizing multiple views without known camera poses. This method estimates all camera poses and objects within the scene across different views and then employs bundle adjustment to optimize the estimated poses.

Certain studies utilize object-level SLAM to estimate the pose of objects [29,30]. Some methods utilize known camera poses, requiring only the prediction of objects’ poses [31,32]. Merrill proposes an object-level SLAM pose estimation method for both symmetric and asymmetric objects, based on semantic keypoints [33]. Li proposes a method that uses an estimated heatmap and detected keypoints from a known-transformation multi-view [34]. However, this approach requires point cloud data for pose refinement, which is unavailable in our work. SyMFM6D introduces a novel technique that employs multiple perspectives for accurately estimating object poses, as described in reference [35]. This method utilizes RGB-D data and combines predicted keypoints with instance segmentation to effectively address challenges associated with symmetric objects.

Yang proposes a two-stage method to estimate pose from multi-view, initially focusing on the translation, followed by the rotation part [12]. Other work views objects in the same scene and uses some method to join the objects in a consistent coordinate system. Other studies have approached pose estimation by viewing objects in the same scene and aligning them within a consistent coordinate system. Although these methods, which rely solely on RGB images, can enhance pose estimation, they encounter challenges related to object scales and measurement uncertainties. Furthermore, some methods concentrate on scenes containing multiple object classes rather than instances of the same class [36]. However, our work introduces a method that addresses these issues effectively.

The proposed approach utilizes known transformations between camera viewpoints to convert all estimates into the world frame. Moreover, this method fuses estimates from various views based on the confidence score of each pose, enhancing the robustness of pose estimates under scenarios involving occlusion and reflective metal parts.

## 3. Methods

**Figure 2:**
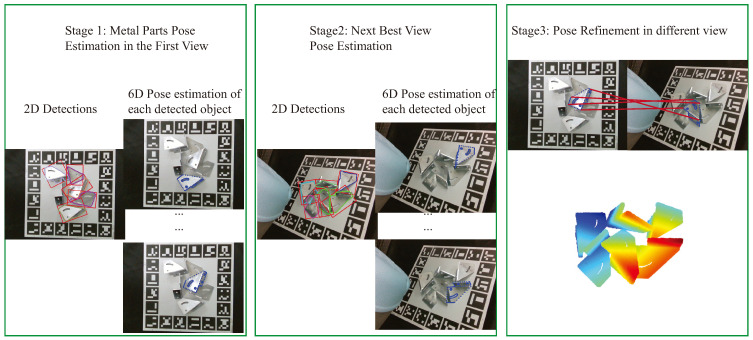
The overview of the proposed multi-view metal parts pose estimation method. In the first stage, this work detects metal parts and estimates the pose of each detected object. The red bounding boxes show the detection results. Then, the proposed method calculates the next best view based on the ray tracing and drives the camera mounted on the arm to the perspective. This work calculates the pose of the metal parts in this view. In the last stage, the proposed method combines the known camera poses and the estimated pose of metal parts in each view to refine the global scene.

Compared with previous work, the proposed approach incorporates a multi-step pipeline, with principal innovations including single-view pose estimation and the use of ray casting to determine the next best view, as illustrated in Figure 2. This work aims to estimate accurate 6D poses of multiple metal parts in RGB images captured with a single camera from multi-view. This work can get a scene with the known pose of metal parts by using known CAD model information and jointly estimating the pose of metal parts in multi-view. This work proposes a strategy to handle the above issues. This work proposes a method to address these problems. First, this work employs a method to estimate the pose of detected metal parts from one view. Second, this work uses ray casting to simulate different views to calculate the next best view, and this mechanism is used to drive the camera to move to the next best view. Third, estimating metal parts in a single view may have some errors. The proposed method needs to find a way to remove the error estimation. Considering this issue, this work uses the confidence score of the corresponding keypoints to judge the error. This work aims to accurately estimate the 6D poses of multiple metal parts in RGB images captured from multiple views using a single camera, leveraging known CAD model information to achieve an accurately posed scene. The proposed method addresses several challenges: first, it estimates the pose of detected metal parts from a single view; second, it employs ray casting to simulate different views and determine the next best view, guiding the camera’s subsequent movement; third, it identifies and addresses errors in single-view pose estimations by using the confidence scores of corresponding keypoints.

### 3.1. Approach Overview

This section outlines a framework for the pose estimation of metal parts from multiple views, aiming to reconstruct a scene with accurately estimated poses. In this scenario, the 3D model of the metal parts is considered known. Challenges such as occlusion or parts being out of view in some perspectives require additional views for further pose estimation, requiring efficient planning of the next viewpoint. The proposed method comprises three main steps, which are summarized in Figure 2.

In the first stage, this work detects metal parts and predicts the semantic keypoints of the corresponding detected metal parts. Subsequently, the proposed method estimates the 6D pose of each detected metal part. However, inaccuracies may arise in these estimated poses, and some parts may fall out of view. Consequently, an additional view is necessary for further refinement of the estimations. The second stage, known as next best view planning, builds on the initial pose estimations of each detected metal part and their corresponding confidence levels, derived from the heatmap values of the semantic keypoints. In this stage, the CAD model of the well-estimated objects is voxelized, and the synthetic camera view is simulated to rotate to identify potential viewing angles. The perspective that reveals the largest newly added visible area, indicating a significant difference from the current view and providing the most complementary information, is selected as the next best view. The final stage of the proposed methodology involves merging and refining the estimated poses from each viewpoint. Given the known camera pose determined through hand–eye calibration, this method integrates the poses of metal parts from various camera orientations. This presents a complex challenge, as the pose estimated from a single view may contain errors.

### 3.2. Single View Pose Estimation

Multiple studies have explored using networks for pose estimation from a single viewpoint. In this section, our proposed method also focuses on a single viewpoint. This approach primarily involves detecting object instances and predicting semantic keypoints. Given an RGB image, the method detects metal parts and estimates their corresponding semantic keypoints. Finally, the strategy outputs the pose of these metal parts with respect to the viewpoint.

The proposed method builds on HRNet [19] to detect semantic keypoints. When using the HRNet to detect keypoints, this work uses 50,000 synthetic images for training and 10,000 synthetic images for validating. Concerning the selection of keypoints, this work chooses the keypoints from the CAD model heuristically. Some metal parts choose the sparse edge points as the keypoints; some choose the sparse points along the symmetry axis as the keypoints, and others choose vertices as the keypoints. When training with the synthetic images, this work sets the batch size as four and trains 20 epochs. The implementation method can be transferred to other types of metal parts since the method can detect kinds of metal parts and predict the keypoints. If given the CAD model of the corresponding metal parts, the pose can be easily estimated. The network operates on cropped images extracted from the detected part. Then, the network outputs the values corresponding to the keypoints.

This work uses synthetic data because of the complexity of annotating keypoints in real-world data, and accuracy cannot be guaranteed. The synthetic data are accessible and can be labeled accurately. The proposed method randomly samples several metal parts from the known CAD models considered, places them randomly, and samples the orientation and position of each object randomly. The images are generated by running the physics simulation Blender 2.79 (Blender Foundation, Amsterdam, Netherlands). for a few seconds using domain randomization [37], generating physical object configurations. The images are photorealistic. In addition, this work sets the object model to have a metal attribute during rendering so that the rendered metal parts appear metallic. The camera is set to point in a specified orientation and position. The rotation angle of metal parts is sampled between $\left(-180^{\circ },180^{\circ }\right)$. Our training dataset is exclusively generated using CAD models. Additionally, this work applies data augmentation techniques to the training dataset, incorporating flipping, brightness adjustment, and Gaussian noise. The proposed system only uses one camera, and this work set the camera’s intrinsic parameters to K. In each view, the work obtains a set of object detections using an object detector and a set of semantic keypoints estimation using a keypoints estimation network. The proposed method uses the PnP algorithm and Ransac [38] to acquire the metal parts pose.

### 3.3. The Next-Best-View Planning

Perspective selection is becoming an increasingly important area in computer graphics. An ideal view should capture maximum details of the metal parts in the current scene, as the quality of this view can significantly influence the results [39].

Randomly sampling possible camera poses may be inefficient. Therefore, it is essential to develop a method that identifies more efficient camera poses. To compute the best view from the camera, the work uses Open3D to render the metal parts model with a resolution of 640×480 pixels without any background. To show the goodness of the viewpoint quality measures, the work uses the number of newly added hit points by ray tracing to judge the view. The simulated perspective containing the maximum number of additional hit points means the perspective contains the most different information from the current perspective. Therefore, this work selects this perspective as the next best view.

The proposed method uses the semantic keypoint heatmap as the probability to judge whether the object is estimated well. After detecting the metal parts and predicting the corresponding keypoints, this work calculates all the predicted confidence scores of the metal parts. The proposed work regresses the heatmaps simply from the high-resolution representations output. In this keypoints prediction network, the loss function is the mean squared error, which compares the predicted and ground truth heatmaps.

The higher the predicted score, the better the pose estimation quality. A low confidence score may imply inaccurate pose estimation. Therefore, this work sets a threshold and only considers cases where the total confidence score $score=\sum_{i=0}^{n}k_{i}$ for the predicted keypoints of each detected object exceeds the threshold.

This work infers the next best view according to the current poses when obtaining the detected object poses and the corresponding possibility of the pose. The distance between the camera and the objects remains unchanged. However, the simulated camera is not fixed. It rotates around the objects at intervals of 5 degrees around the x-axis, at 5 degree intervals around the y-axis, and at 10 degree intervals around the z-axis. The proposed method uses the model of metal parts and pose relative to the arm base to make the scene voxelization. By employing the ray casting in Open3D, this work generate rays and 6D vectors with origin and direction. When this work voxelizes the metal parts, the relative pose between objects and arm base is fixed. This work uses $P_{base2obj}=P_{base2hand}\times P_{hand2cam}\times P_{cam2obj}$ to obtain the relative pose. The hand represents the gripper fixed in the arm. The $P_{base2obj}$ represents the pose of the object frame relative to the arm base frame. The $P_{base2hand}$ represents the pose of the hand frame relative to the arm base frame. The $P_{hand2cam}$ represents the pose of the camera frame relative to the hand frame. The $P_{cam2obj}$ represents the pose of the object frame relative to the camera. Since their relationship is fixed, the method can acquire the index of each voxel, which is solid. This work employs ray tracing to get the index of the hit triangle meshes.

**Figure 3:**
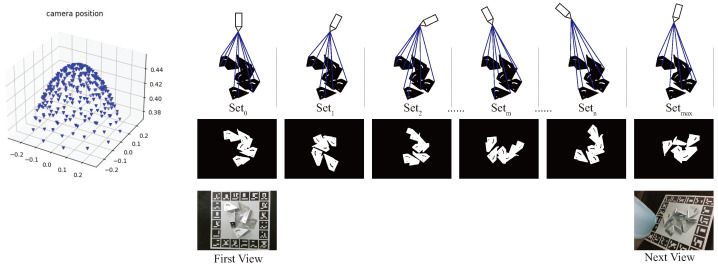
The way of calculating the max area. This work calculates all the hit points in the first view $set_{0}$, rotates the camera around the voxel, and calculates the number of hit points $set_{i}$. The simulated camera is not fixed. It rotates around the objects at intervals of 5 degrees around the x-axis, at 5-degree intervals around the y-axis, and at 10-degree intervals around the z-axis. The voxel in the first view is the metal parts with the score of pose estimation above the threshold. By calculating the additional hit points between new view and the first view $add_{i}=set_{i}-\left(set_{i}\&set_{0}\right)$, this work can get the next best view.

The proposed method employs ray casting to compute ray intersections with meshes. If the ray hit the mesh, it means the corresponding voxel is visible. By counting the number of meshes being hit, this work can obtain the visible area of this view. The voxel size is 1 mm. This work meshes the metal parts models with the estimated pose. As shown in Figure 3, this work uses the number of hit voxels to represent the visible area and employs Equation (1) to calculate the number of addtional hit points. The $set_{i}$ represents the number of hit voxels and the voxel’s corresponding index IDs of the synthetic view *i*. The proposed method calculates the maximum additional area by changing the relative poses between the virtual camera and the object pose coordinates as shown in Equation (2). The $\sum_{Conf>thre}P_{base2obj}^{k}$ is the combination of objects whose pose estimation confidence is larger than the pose threshold. Equation (2) calculates the maximum additional hit points between the kinds of views and the first view. Then, this work can get the next best view according to the parameter and the corresponding camera pose.

(1) $$add_{i}=set_{i}-\left(set_{i}\&set_{0}\right)$$

(2) $$\max_{i}\left(set_{i}\left(\sum_{Conf>thre}P_{base2obj}^{k}\right)-set_{0}\left(\sum_{Conf>thre}P_{base2obj}^{k}\right)\right)$$

After the work gets the next best view, the proposed method drives the robot arm to the subsequent best viewpoint of the scene. Because the relative poses between objects and the arm base are fixed, the work can quickly obtain the camera pose for the next view. Using hand–eye calibration, the work can get the end-effector pose and drive the arm to set the camera to the specified pose.

When calculating the arm pose for the next best view, this work must consider whether the robot arm can reach the pose. Take the UR5e for example: the UR5e can reach 850 mm. The proposed work employs the desired pose of six degrees of freedom in the arm and the DH parameters of the robot arm to find the set of joint configurations $Q=\left\{q_{i}\right\}$ where $q_{i}=\left(\theta_{i}^{1},\theta_{i}^{2},\theta_{i}^{3},\theta_{i}^{4},\theta_{i}^{5},\theta_{i}^{6}\right)\in {\left[0,2\pi \right)}^{6}$ that satisfies the desired position and orientation of the final link.

### 3.4. Scene-Level Pose Refinement

The proposed method makes the pose of the camera relative to the world frame known, which is known as $T_{wc}\in SE\left(3\right)$. This work installs the sensor on the robotic arm. This work can obtain the relative pose between the camera and the robot arm’s end effector by employing the eye-in-hand calibration method [40]. The proposed method aims to refine the metal parts’ poses from different perspectives in the world frame $T_{wo}\in SE\left(3\right)$.

**Figure 4:**
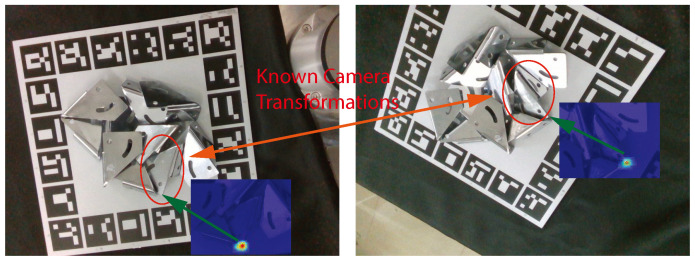
The information from multiple viewpoints is integrated to estimate an object’s 6D pose. Using keypoint heatmaps from individual views, this work calculates multi-view uncertainty estimates. The image with green arrow indicates the heatmap of the corresponding keypoint. The red arrow shows the corresponding metal part in different views.These estimates are then used for filtering and ranking candidate poses, demonstrating improved accuracy and reliability compared to existing methods.

The metal parts are $P_{1}^{a},P_{2}^{a},\ldots ,P_{k}^{a}$, in which *k* is the number of detected metal parts, and *a* is the viewpoint. This work chooses semantic keypoints of higher confidence scores with corresponding objects when using multiple views. The last step aims to employ the objects’ estimated pose in the corresponding view and related camera pose to retrieve the scene model of objects, as shown in Figure 4.

In detail, this stage estimates metal parts’ poses in different camera coordinates in order to combine the poses in a common coordinate frame. The camera is fixed in the robot arm. By using a hand–eye calibration method, this work can obtain the pose between the robot arm end and the camera, which is represented as $P_{hand2arm}$. The poses between objects and the robot arm base are solid. They are expressed as $P_{base2obj_{i}}=P_{arm_{V}}\;\cdot \;P_{hand2cam}\;\cdot \;cam_{V}2P_{obj_{i}}$. The $P_{obj_{i}2base}$ is the pose between the *i*th object and the robot base, which is solid. The $P_{arm_{V}}$ is expressed as the pose between robot arm end and the robot arm base in the *V*th view. The $cam_{V}2P_{obj_{i}}$ is expressed as the pose between the *i*th object and the camera in the *V*th view. The $P_{arm_{V}}$ is known according to the robot arm interface. The $cam_{V}2P_{obj_{i}}$ is calculated through the confidence score of the keypoints and the pose in each view. This work calculates the confidence score of semantic keypoints about the *i*th object in each view and chooses the best score of the *i*th object and the corresponding view $V_{best_{i}}$. The pose between the *i*th object and camera $cam_{V}2P_{obj_{i}}$ is $cam_{V}2P_{obj_{i}}=inv\left(P_{arm_{V}}\;\cdot \;P_{hand2cam}\right)\;\cdot \;P_{arm_{V_{best_{i}}}}\;\cdot \;P_{hand2cam}\;\cdot \;cam_{V_{best_{i}}2P_{obj_{i}}}$. In is way, the proposed method can obtain the metal parts from different views in the world coordinate frame.

## 4. Experiments

In this section, this work evaluates the proposed method in the setup experiments. First, this work validates and analyzes metal parts with single-view pose estimation results. This work notably verifys that the proposed approach performs well. Subsequently, this work validates the proposed pose estimation strategy with multiple viewpoints.

### 4.1. The Ground Truth Poses

The reflective properties of metal parts can introduce inaccurate depth maps, and the RGB images can have different textures and fake edges, particularly in cluttered scenarios. To assess the performance of the pose estimation method, this work employs an industrial-grade camera to capture the ground truth depth. The industrial-grade depth cameras often fail to sense complete depths when surfaces are too glossy or dark, or are too close to or far from the sensor. In order to capture complete depth maps of metal parts, we use the anti-reflective scanning spray on objects.

**Figure 5:**
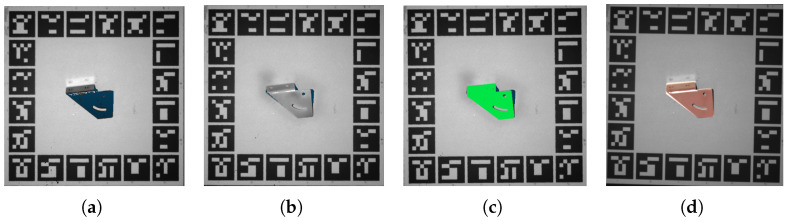
Illustration of using scanning spray to determine the pose of metal parts relative to an industrial-grade camera. (**a**) Image of metal parts without scanning spray. (**b**) Image of metal parts with scanning spray applied. (**c**) Selected keypoints are used to align the point cloud with the model and obtain the ground truth pose. (**d**) The rendered color indicates the estimated pose obtained through the proposed pose estimation method, captured using a Realsense camera.

The scanning spray generates a homogeneous layer with only 8–15 ηm thickness, which satisfies the expected depth accuracy. The spray will self-evaporate within four hours. Figure 5b shows the captured depth map after using the scanning spray. Compared with the captured depth map without spray (Figure 5a), the industrial-grade sensor can acquire complete depth maps with less noise when the objects are covered with scanning spray.

After obtaining the metal parts’ point cloud data, this work uses the point cloud registration to get the pose of metal parts relative to the industrial-grade camera. The proposed method first segments the cloud points of all the metal parts individually. This work uses Meshlab to remesh the CAD model and chooses some metal parts’ keypoints to align the point cloud with the CAD model. Finally, the proposed method uses the ICP (iterative closest point) to refine the pose, as shown in Figure 5c. Figure 5d portrays the pose estimation result of the metal parts in the Realsense camera.

### 4.2. Single-View Pose Estimation Experiment

**Figure 6:**
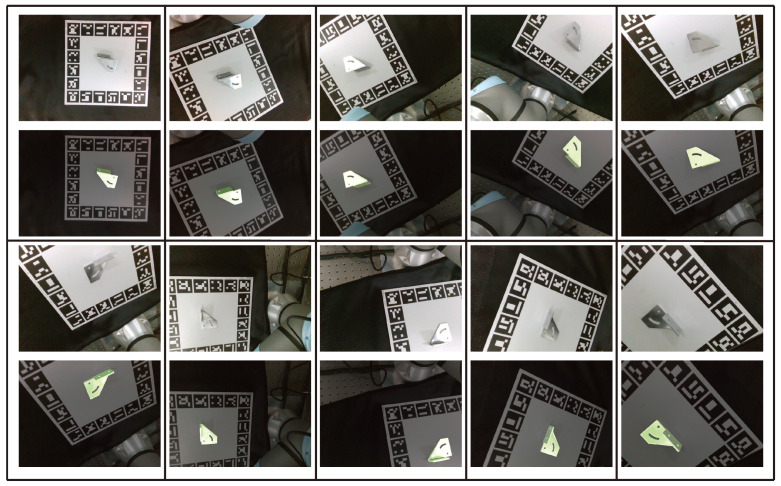
The qualitative results pose estimation for a single metal part from a single view. Each scene is organized into two rows: the first row shows the raw image, and the second row presents the rendered pose of the object.

The qualitative results of pose estimation from a single view are shown in Figure 6. Because the relative pose between the metal parts and the Aruco marker board is fixed, this work sets the relative pose captured by this high-end camera as the ground truth, and the relative pose captured by the Realsense camera as the estimated pose.

The proposed method uses $P_{Hm2o}=P_{Hm2c}\;\cdot \;P_{Hc2o}$ to calculate the pose of objects relative to the marker board in the high-end camera, and uses $P_{Rm2o}=P_{Rm2c}\;\cdot \;P_{Rc2o}$ to calculate the pose of objects relative to the marker board in Realsense sensor. This work uses Equation (3) to measure the difference between the ground truth pose and the estimated pose. In the rotation part, this work converts the matrix to angle-axis format and uses the angle to judge the difference. In the translation part, this work uses the Euclidean norm to represent the difference. In the experiments, the angle error of the rotation part is $4.09^{\circ }$; in the translation part, the error is 4 mm, and the accuracy is 91.17%.

(3) $$\begin{array}{cc} ▵\theta & =arccos(\left(trace(R_{i}\;\cdot \;R_{gt}^{T})-1\right)÷2)\\ ▵T & ={∥T_{i}-T_{gt}∥}_{2} \end{array}$$

**Figure 7:**
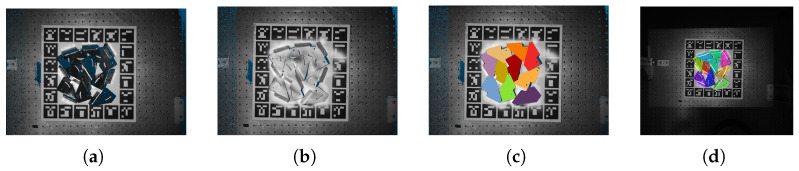
Illustration of using scanning spray to determine the pose of multiple metal parts relative to an industrial-grade camera. (**a**) Point clouds of the metal parts without scanning spray. (**b**) Point cloud of the metal parts coated with scanning spray. (**c**) Manual segmentation of the point cloud for each metal part. (**d**) Alignment of the point cloud using keypoints to obtain the ground truth pose. The color overlaid in this image represents the ground truth pose.

In the multiple metal parts pose estimation section, this work uses the method shown in Figure 7 as the ground truth. We use Equation (4) to evaluate the multiple metal parts pose estimation approach. The $R_{gt}$ and $T_{gt}$ represent the ground truth for rotation and translation, respectively, whereas $R_{est}$ and $T_{est}$ refer to the estimated results. In this work, the constant $k_{m}$ is set to 0.1. The variable *d* represents the diameter of the metal parts. The expression x∈M denotes all points x within the model *M*. In our test, our pose estimation accuracy can reach 82%.

(4) $$m=\underset{x\in M}{avg}∥\left(R_{gt}x+T_{gt}\right)-\left(R_{est}x+T_{est}\right)∥$$

**Figure 8:**
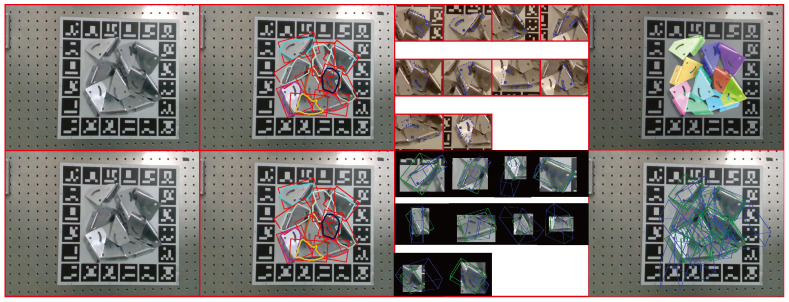
The images illustrate the pose estimation results of multiple metal parts from a single view. The first column displays the raw image, and the second column shows the target detection results. The third column in the first row presents the predicted semantic keypoints for each target, and the last column in the first row displays the rendered pose of these targets. In the second row, the third column depicts the estimated pose of each metal part using PVNet, and the last column presents the estimated pose of all targets. Green 3D bounding boxes indicate the ground truth, and blue bounding boxes represent the estimated poses. The comparison results demonstrate that our method surpasses PVNet in estimating the poses of shiny metal parts.

Figure 8 compares the qualitative results of metal parts pose estimations between our proposed method and PVNet [11]. PVNet employs the same synthetic images for training both the keypoints and object pose. When using PVNet to predict the keypoints, we utilize the same object detection network to identify the metal parts.

### 4.3. Multi-View Experiments

In the single-view experiments, the pose estimation method demonstrates good results. Subsequently, this work evaluates the performance of the multi-view metal parts pose estimation.

**Figure 9:**
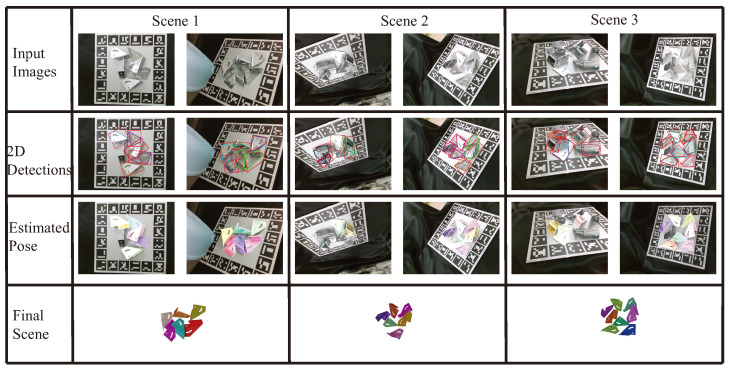
The qualitative results outlines the multi-view pose estimation process for metal parts. The first row displays input RGB images from various viewpoints. The second row illustrates the results of 2D detection of metal parts. The third row depicts the rendered estimated poses for the current view, noting that objects without rendering indicate a low confidence score in their estimation. The fourth row presents the final pose refinement. In each scene, the second column shows the subsequent perspective and corresponding estimation results of the first column. The red dounding boxes are the detection results. By employing the next-best view planning method, the proposed method is able to identify more estimated objects compared to using only the first view.

First, to minimize gross errors, the proposed method only considers the detected metal parts with good detection scores, which are higher than 0.9. Then, this work calculates the pose of detected and considered metal parts. For the multi-view metal parts pose estimation work, this work uses one GeForce RTX 2080 (NVIDIA Corporation, Santa Clara, CA, USA) as the Graphics Card for training. The proposed method takes roughly 1.3 s for 2D metal parts detection per view. In the semantic keypoints part, it takes about 1.3 s. Obtaining the pose with the PnP and RANSAC methods incurs a processing time of 0.4 s. The qualitative results are illustrated in Figure 9. When driving the arm to the calculated next-best view, the proposed method can estimate more objects compared to using only the initial view.

**Figure 10:**
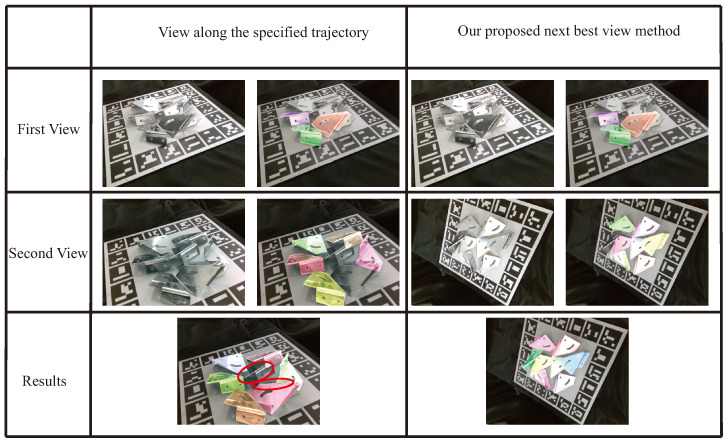
The comparision results present examples of comparison results. The first column displays the specified camera trajectory used to guide the robot arm, and the second column shows the corresponding estimation results, overlaid with colorfully rendered poses. The third column demonstrates how the camera was moved to the next best view using the proposed method, and the fourth column presents the outcomes of our proposed method, overlaid with colorfully rendered poses. The last row reveals the final pose refinement results after employing two different moving camera methods for two views. The red curves from the last row in the specified trajectory method indicate the metal parts that did not achieve pose estimation. The figure illustrates that the proposed method can estimate more metal parts than the specified trajectory method.

This work compares the pose estimation results using different next views, with the comparison results showcased in Figure 10. The first column displays a specified camera trajectory driving the robot arm along an axis of approximately 15 mm, followed by the corresponding estimation results in the second column. The third column illustrates our proposed method’s capability to drive the camera to the next best view, with the fourth column presenting the results of this method. The figure demonstrates that our method can adaptively select views that offer more information. The last row reveals the final pose refinement results after employing two different moving camera methods for two views. The estimation results indicate that our next-best-view planning method outperforms the specified trajectory method, with the ability to estimate more metal parts after two views.

### 4.4. Grasping Experiments

**Figure 11:**
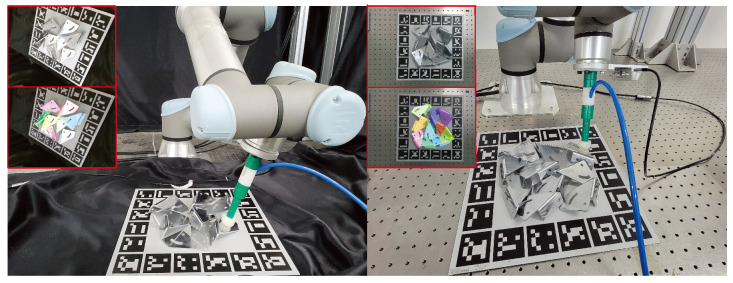
Examples of pose estimation and grasping experiments. When estimating all the metal parts’ poses, this work drives the robot arm to grasp the object. The upper left corner is the raw image taken from the camera and the rendered objects with estimated poses.

In order to verify the accuracy of pose estimation, this work conducted grasping experiments. The proposed method uses a UR5e robot arm (Universal Robots, Odense, Denmark) equipped with an Intel Realsense R435 camera (Intel Corporation, Santa Clara, CA, USA) to grasp metal parts according to the estimated pose. After obtaining all the metal parts’ poses, the proposed method drives the arm to grasp according to the estimated pose. This work conducted the grasping experiments with multiple metal parts scenarios. The success rate is 92% when it takes 50 times to grasp metal parts. As shown in Figure 11, this work can accurately estimate the poses and grasp the metal parts successfully.

## 5. Conclusions

The main contribution of this paper is the proposal of a novel method for calculating the next best view in pose estimation using a single camera. The novelty of this paper lies in the use of ray tracing to calculate the additional view area from various virtual cameras. This approach allows us to determine the next best view, which contains significantly more distinct and valuable information. The next best view enables an approach to accurately recover the 6D pose of multiple metal parts from various viewpoints. A key advantage of this method over existing approaches is its use of ray tracing to calculate the most informative next view of the scene. This work utilizes semantic confidence scores of keypoint estimates to enhance the reliability of pose estimation by efficiently discarding poses with higher errors. Additionally, the proposed method does not require depth measurements, making it particularly suitable for metal parts. Experimental results demonstrate the robustness of the metal parts pose estimation using multi-view RGB images with a camera of known pose.

## Data Availability

All the available data are reported within this paper.
